# 

**Published:** 1883-03

**Authors:** 


ORIGINAL COMMUNICATIONS:
Medical Clinic. Alfred L. Loomis, M.D. .......... 129
The Cause of Caries—Bacteria or Acids? F. Y. Clark, M.D., D.D.S. .......... 132
Per-Oxide of Hydrogen ($H^2$ $O_2$). A. W. Harlan, D.D.S. Chicago .......... 138
Practical Hints for Dentists. J. A. Robinson, D.D.S., Jackson, Mich. .......... 139
ORIGINAL TRANSLATIONS AND ABSTRACTS:
An Operation for Pyonephrosis .......... 142
Abstract of Discussion of the Treatment of Deciduous Teeth .......... 146
SELECTIONS:
Remarks on the Pathology and Treatment of the Pneumonia of Early Infancy .......... 152
The Close Relation Existing Between, and Probable Identity of, Scarlatina and Diphtheria .......... 160
Perinephritic Abscess .......... 165
The Effects of Division of the Vagi Upon the Heart. A. M. Bleile, M.D., and A. Feil, Columbus, O. .......... 167
EDITORIAL:
The Use of the Gum Lancet .......... 170
Pasteur's Experiments Concerning Contagious Diseases .......... 173
Operations for Removal of the Kidney .......... 174
Etiology of Dental Caries .......... 175
A Convenience .......... 175
Platinum and Gold Blocks .......... 176
CURRENT NEWS AND OPINION:
New York State Medical Society .......... 176
The Cartwright Lectures .......... 177
New Remedy in Diphtheria .......... 178
Intestinal Obstruction Relieved by Massage .......... 178
Aspiration of a Serous Fluid in the Pericardium. Recovery .......... 179
BIBLIOGRAPHICAL .......... 180
OBITUARY:
Marshall H. Webb, D.D.S. .......... 185
Lafayette Ranney, M.D. .......... 186
Mrs. Elizabeth A. Beard .......... 186
EXTRACTS:
Polio-(Trepho)-Myelitis Anterior .......... 187
Thomas on Extra-Uterine Pregnancy .......... 188
Addison's Disease .......... 188
POPULAR SCIENCE DEPARTMENT:
The Discovery of Grape Sugar .......... 189

